# New 3D Ink Formulation Comprising a Nanocellulose Aerogel Based on Electrostatic Repulsion and Sol-Gel Transition

**DOI:** 10.3390/polym17081065

**Published:** 2025-04-15

**Authors:** Qing Yang, Haiyang Yu, Xiaolu Wang, Yunze Li, Dan Li, Fu Guo

**Affiliations:** 1 College of Materials Science and Engineering, Beijing University of Technology, Beijing 100124, China; yangqing@emails.bjut.edu.cn (Q.Y.); yuhaiyang@emails.bjut.edu.cn (H.Y.); wangxiaolu2019@bjut.edu.cn (X.W.); liyunze18612869603@163.com (Y.L.); guofu@bjut.edu.cn (F.G.); 2 Key Laboratory of Advanced Functional Materials, Education Ministry of China, Beijing University of Technology, Beijing 100124, China; 3 School of Mechanical Electrical Engineering, Beijing Information Science and Technology University, Beijing 100192, China

**Keywords:** cellulose, direct ink writing, aerogel, freeze drying

## Abstract

New 3D printing aerogel materials are environmentally friendly and could be used in environmental protection and biomedical fields. There is significant research interest in 3D printing cellulose-based aerogels since cellulose materials are biocompatible and are abundant in nature. The gel-like nature of the cellulose water suspension is suitable for 3D printing; however, the complexity and resolution of the geometry of aerogels are quite limited, mainly due to the inks’ low viscosity that fails to maintain the integrity of the shape after printing. To address this limitation, a carefully optimized formulation incorporating three key ingredients, i.e., nanofibrils (TEMPO-CNFs), 2,2,6,6-tetramethyl-1-piperidinyloxy modified cellulose nanocrystals (TEMPO-CNC), and sodium carboxymethyl cellulose (CMC), is utilized to enhance the viscosity and structural stability of the ink. This combination of cellulose derivatives utilizes the electrostatic repulsive forces between the negatively charged components to form a stable and uniformly distributed suspension of cellulose materials. Our ink formulations improve printability and shape retention during 3D printing and are optimal for DIW printing. We print by employing an all cellulose-based composite ink using a modified direct ink writing (DIW) 3D printing method, plus an in situ freezing stage to form a layer-by-layer structure, and then follow a freeze-drying process to obtain the well-aligned aerogels. We have investigated the rheological properties of the ink formulation by varying the concentration of these three cellulose materials. The obtained aerogels exhibit highly ordered microstructures in which the micropores are well-aligned along the freezing direction. This study demonstrates a strategy for overcoming the challenges of 3D printing cellulose-based aerogels by formulating a stable composite ink, optimizing its rheological properties, and employing a modified DIW printing process with in situ freezing, resulting in highly ordered, structurally robust aerogels with aligned microporous architectures.

## 1. Introduction

Direct ink writing (DIW) is an extrusion-based additive manufacturing (AM) technology that builds 2D or 3D structures through extruding materials in a layer-by-layer manner [1,2,3]. This technique has gained considerable attention because of its extensive material adaptability, which allows for the creation of complex geometries [4,5]. Its advantages include a diverse range of material options (such as hydrogels, ceramics, polymers, etc.), precise control over micro-scale geometry, and relatively simple equipment requirements [6]. For example, H. Yuk et al. designed a semi-quantifying 3D printing strategy through analyzing printed objects by modifying printing parameters to improve the printing quality [7]. The DIW process can be divided into three stages: extrusion, solidification, and layer support [8]. These stages are critical for determining the final print quality, as each step influences the material behavior and structural integrity of the printed objects. Effective control of these material properties during extrusion and solidification is essential for maintaining the desired print quality and ensuring precise layer formation. By regulating the thixotropic and shear-thinning properties of materials through the control of rheological attributes to optimize the printability of inks, we can ensure the production of high-resolution printed objects using DIW technology [9]. The rheological properties of inks are often determined by factors such as molecular weight, concentration, temperature, and chemical and physical interactions (e.g., hydrogen bonding, electrostatic attraction and repulsion, hydrophilicity/hydrophobicity, and van der Waals forces) [10,11,12]. Direct ink writing technology faces limitations in aspects such as relatively low printing precision and speed, complex equipment and process requirements, potential nozzle clogging, the need for support structures, and restricted material selection and performance consistency. Thus, optimizing the rheological properties of inks is crucial for achieving the high resolution DIW 3D printing of cellulose materials.

In recent years, as a sustainable and highly functional natural material, nanocellulose has been extensively used in DIW printing for the fabrication of functional composites [13,14]. Cellulose, the most abundant and widely distributed renewable polysaccharide on Earth [15], contains numerous hydroxyl groups, which enable it to be modified and functionalized under various physical and chemical conditions [16]. The rheological properties are closely related to the printability of the composite inks during the DIW printing process, and the printed models match the digital design. From a rheological viewpoint, an ink must flow smoothly without clogging the nozzle outlet or dripping, meaning that shear thinning behavior and proper viscosity are the most important parameters for producing smooth DIW print. Cellulose-based inks exhibit shear-thinning behavior and are colloidal forms fit for DIW deposition [17,18]. Once extruded from the nozzle, the ink transitions from the shear-thinning gel to a solid-like paste to maintain its shape for the layer-by-layer printing [3]. One promising approach for improving the printing resolution and scaffold structure in DIW printing is the use of cellulose nanofibers (CNFs), which display good mechanical strength and can be well dispersed in most aqueous media. Many CNFs composites have been used in DIW printing, while scaffolds with well-ordered structures and high printing resolution have yet to be achieved [19]. This limitation primarily arises from the difficulty in balancing the flowability and shape retention of the material during the deposition process [20], which has hindered the precise control of the scaffold arrangement and microstructure. Developing modified CNFs inks with higher adhesiveness and more precise rheological properties is crucial for further enhancing the printing resolution and functional characteristics of the structures [21]. The use of DIW to print customizable 3D structures from a single-component formulation of 2,2,6,6-tetramethyl-1-piperidinyloxy modified cellulose nanofibers (TEMPO-CNFs) has been explored [22]. Single-component formulations of cellulose nanofibers (TEMPO-CNFs) do not exhibit high print quality and shape fixation in DIW 3D printing, and the current printing resolution remains at ~300 μm, with rough printed layer structures [23,24]. Tong, Y., and Mariani, L. M., found that CNFs and cellulose nanocrystals (CNC) can enhance the printing performance and product quality of CNFs-based inks by increasing the viscosity and yield stress, improving the printing continuity and precision [25,26]. In any case, due to the limitations of a single type of cellulose in rheological optimization, the resolution of the printed structures remains relatively low (100–500 μm).

In this work, we develop a novel composite ink formulation and adjust its rheological properties by varying the inks’ concentrations to achieve high-precision 3D printed structures. Since all three materials are negatively charged and dispersed in water media, they can partially mitigate molecular entanglements and hydrogen bonding interactions with each other without forming sediments [27]. In addition, this combination of materials can modulate the rheological behavior during the printing process, making it more suitable for the molding needs of complex geometries while providing higher support and solidification [28]. This work demonstrates that the optimized composite ink formulation exhibits optimal rheological properties in DIW printing under the influence of multi-scale coordination and intermolecular interactions. Composite cellulose aerogels with high printing quality and shape fixation (around 100 μm) can be achieved with the aid of an in situ freezing step during the printing process. DIW printing and directional freeze-drying enables the fabrication of cellulose aerogels with aligned micropores and multilayered, interconnected architectures, offering promising potential for advanced biomedical applications.

## 2. Materials and Methods

### 2.1. Materials

**Scheme 1:**
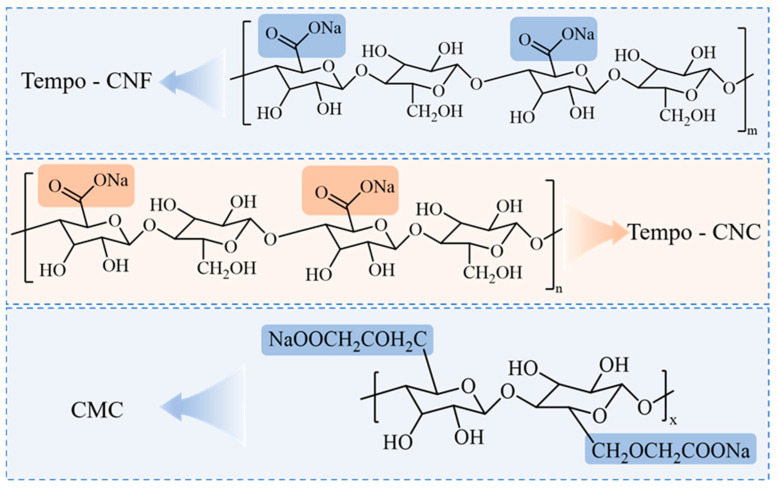
The molecular structure of the three types of cellulose materials used in forming the cellulose composite ink.

Scheme 1 illustrates the three kinds of cellulose materials used for preparing the composite ink. In detail, we blended three components, i.e., TEMPO-CNFs, 2,2,6,6-tetramethyl-1-piperidinyloxy modified cellulose nanocrystals (TEMPO-CNC), and sodium carboxymethyl cellulose (CMC), to form extrudable inks. The addition of TEMPO-CNC improves the mechanical stability of the material. The addition of CMC as a thickener regulates the viscosity of the composite cellulose and maintains the shape of the material during the printing process. Upon dissolution in water, all three materials exhibit a network of negatively charged properties, where molecular entanglement and hydrogen bonding interactions can be partially relieved by electrostatic repulsive forces. This results in the improved dispersion and rheological properties of the system [29,30,31,32].

An in-situ freezing process has been incorporated into the printing procedure with a view of improving printing quality and shape fixation. This freezing process serves to reduce the shrinkage and deformation of the material post-printing, thereby ensuring the dimensional shape fixation of the printed component.

Finally, freeze-drying is applied to the printed samples. This process leads to the formation of multi-scale pore structures within the aerogel. These pore structures have been shown to significantly increase the specific surface area of the material and improve the adsorption properties of the aerogel.

The main information for the composite ink is shown in Table 1. TEMPO-CNFs suspensions (3 wt%) and TEMPO-CNC suspensions (10 wt%) were purchased from Wood Spirit Biotechnology Co., Ltd. (Tianjin, China) and were diluted in water to the desired concentrations before use. For TEMPO-CNFs, the polymerization degree is 110, and the carboxylate content is 1.86 mmol/g; for TEMPO-CNC, the polymerization degree is 100, the average length is 120 nm, the diameter is 5–10 nm, and carboxylate content is 2.03 mmol/g [33]. Sodium carboxymethyl cellulose was purchased from Aladdin Biochemical Technology Co., Ltd. (Shanghai, China). The 3D printer was purchased from Xiaospear Automation Technology Co., Ltd., (Shenzhen, China). The air compressor (Outstanding 550W-30L) was purchased from Otus Industry and Trade Co., Ltd., (Shanghai, China). The nozzles were purchased from YIDIZHAN Industrial Entilties Co., Ltd., (Dongguan, China).

### 2.2. Methods

#### 2.2.1. Gelation of TEMPO-CNF/TEMPO-CNC/CMC Composite Ink

The total concentrations of the cellulose derivatives are 5 wt%, 4 wt%, and 3.5 wt%, respectively. The formulation of the composite ink is shown in Table 2. Specifically, the 5 wt% formulation requires the combination of 50 g of TEMPO-CNFs suspension, 9 g of TEMPO-CNC suspension, and 1 g of CMC prior to printing. For the 4 wt% formulation, 50 g of TEMPO–CNFs suspension and 9 g of TEMPO-CNC suspension are suspended in 1 milliliter of deionized water (DI water) prior to printing. For the 3.5 wt% formulation, 50 g of TEMPO–CNFs suspension and 1 g of CMC are suspended in 9 milliliters of DI water prior to printing. The various formulations are mixed with DI water to form composite inks, labeled as 5 wt%, 4 wt%, and 3.5 wt% formulations. The 5 wt%, 4 wt%, and 3.5 wt% composite inks are mechanically stirred at 2000 rpm for 3 min to achieve uniform dispersion. Further homogenization is performed using a centrifugation process at 4000 rpm for 5 min. Using the above method, a bubble-free, homogeneous composite ink can be obtained.

#### 2.2.2. 3D Printing of TEMPO-CNFs/TEMPO-CNC/CMC Composite Ink

**Figure 1:**
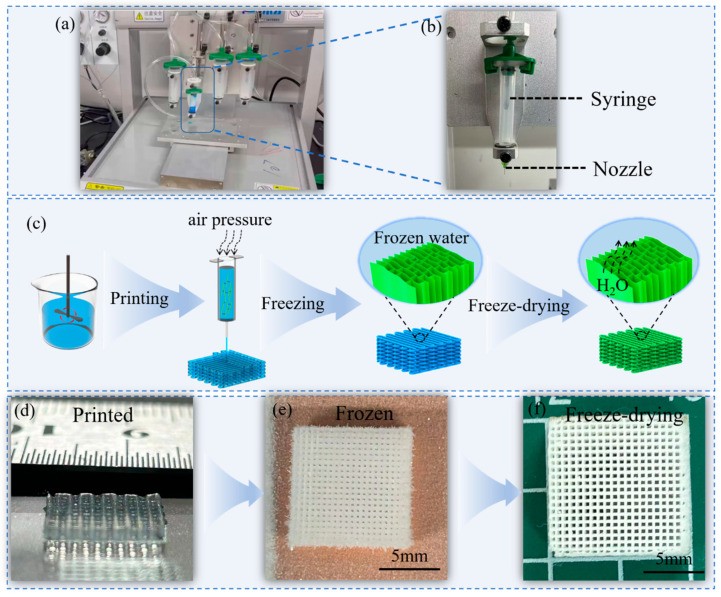
(**a**) Image of the printing set up; (**b**) the printing syringe and nozzle used; (**c**) schematic diagram of the experimental flow of cellulose composite inks for aerogels; (**d**–**f**) overview of the printed composite ink, the printed composite ink after freezing, and the freeze-dried printed microarchitectures.

The prepared composite ink is loaded into a 3 mL syringe, which is then installed at the printing nozzle of the 3D printer. The appropriate printing parameters are set with the nozzle speed ranging from 5 to 100 mm/s, and the distance between the nozzle and the substrate is adjusted from 50 to 200 μm. The composite ink is printed using a 3D printer with a nozzle diameter ranging from 60 to 500 μm and an air pressure of 400 to 600 kPa. The 3D structure is programmed using the built-in controller of the 3D printer. During the printing process, the composite ink is deposited onto a cold Peltier stage (−30 °C) to maintain the shape of the deposited structure after printing. Since the deposited ink can freeze within a few seconds, the structure exhibits excellent fidelity without collapse. The main components of the 3D printer are shown in Figure 1a,b.

#### 2.2.3. Preparation of Nanocellulose Aerogel

The conventional DIW method to prepare aerogels usually consists of the continuous extrusion of printable ink from a nozzle and the layer-by-layer deposition of layers along a designed path, followed by a rapid solid-to-gel transition and drying process [25,34]. In this paper, we performed an in situ freezing (−10 °C) treatment after ink deposition to maintain the ink’s 3D structure. The experimental schematic process of fabricating aerogels is shown in Figure 1c, and the printed aligned composite inks are shown in Figure 1d. Next, the deposited ink was freeze-dried at −30 °C for 24 h to achieve the aerogels (Figure 1e), followed by freeze-drying at −60 °C and below 1 mbar for 48 h (Figure 1f, Table 3). Freezing at −20 °C for 24 h is sufficient to freeze the water into ice. The subsequent steps are sufficient to completely freeze the water in the deposited ink structure, helping the printed structure maintain higher integrity.

### 2.3. Material Characterizations

The equipment and conditions required for the different experiments are shown in Table 4. The zeta potential of TEMPO-CNFs/TEMPO-CNC/CMC was measured using the Zetasizer Nano ZS90, Malvern Panalytical (Shanghai, China). The samples were diluted with DI water to 2.5 wt%/1.5 wt%/1 wt%, respectively. The ratio was kept consistent in the three composite inks. The results are reported as the average of three specimens.

To measure shear viscosity, the shear rate was increased from 1 to 100 s^−1^ at room temperature (25 °C). Step strain experiments were conducted at different shear rates, and the shear rates were maintained for 60 s, changing from 0.1 s^−1^ to 100 s^−1^ and then to 0.1 s^−1^, respectively. Rheological analysis was carried out on the ink formulation using an Austria Anton Paar MCR 302 (Shanghai, China) device, in which a parallel plate (40 mm diameter) was used, with a fixed gap of 1 mm. Rheological analysis of ink formulations was conducted using the Herschel–Bulkley fluid (Shanghai, China) model.

The Herschel–Bulkley fluid model is expressed as shown in Equation (1) [35]:

(1) $$\tau =\tau_{0}+m{\left(\gamma \right)}^{n}$$

where *τ* and *τ*_0_ are apparent stress and static shear stress, *γ* is shear rate, and m is the consistency index.

The flow behavior can be summarized according to the methods in Refs. [36,37,38]:

When n > 1, the fluid exhibits shear thickening behavior.

When n = 1, the fluid behaves like a Newtonian fluid.

When n < 1, the fluid undergoes shear thinning.

Stress-controlled oscillatory amplitude sweeps were measured at a constant frequency of 1 Hz, with shear stress ranging from 1 to 1000 Pa. Scanning electron microscope (SEM) images were obtained using an FE-STEM SU9000 instrument from HITACHI (Shanghai, China) at an operating voltage of 10.0 KV. The compression test was performed using an E43.104 Electronic universal testing machine (Shenzhen, China), with a force sensor of 1000 N. Compression tests were performed on the top surface of the cubic samples. Each sample was compressed at a maximum strain of 90% at a rate of 0.5 mm/min to obtain a stress–strain curve.

## 3. Result

### 3.1. Zeta Potential Analysis

**Figure 2:**
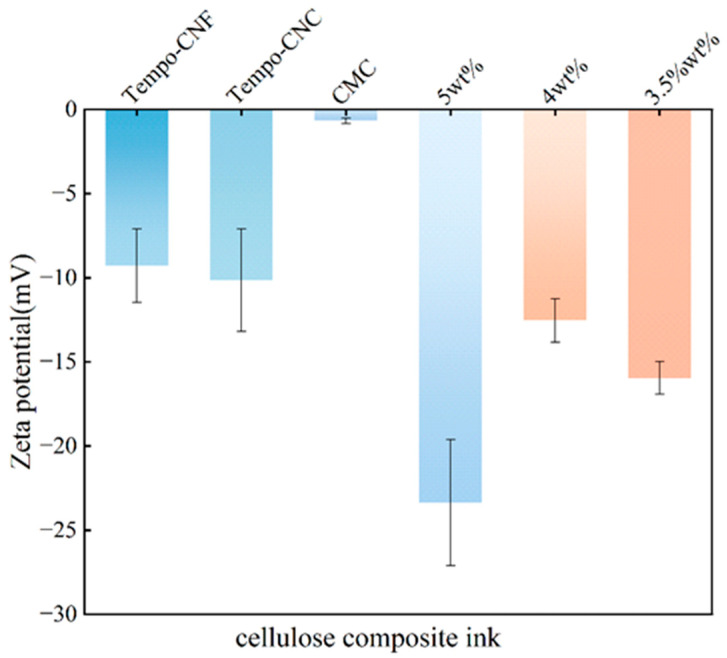
Zeta potential of the individual cellulose materials and the three cellulose composite ink formulations.

The zeta potential of cellulose inks was determined to assess the magnitude of the surface charge of the cellulose particles, which in turn reflects their colloidal stability. As shown in Figure 2, all cellulose materials and inks show negative zeta potential values, meaning that the TEMPO-CNFs, TEMPO-CNC, and CMC materials are negatively charged. The charge density changes resulting from the increasing concentrations of the composite inks suggests that the mixture has a synergistic effect on the formation of negative suspensions. The zeta potential value of the 5 wt% composite ink exhibits the highest absolute values of 23.37 mV compared to those of other two formulations, suggesting that this ink displays good colloidal stability. Thus, the 5 wt% composite ink is more favorable for 3D printing, and we adopt this formulation for the DIW printing demonstrations and for the mechanical testing of the printed aerogels.

### 3.2. Rheological Properties

#### 3.2.1. Viscosity of the Composite Ink

**Figure 3:**
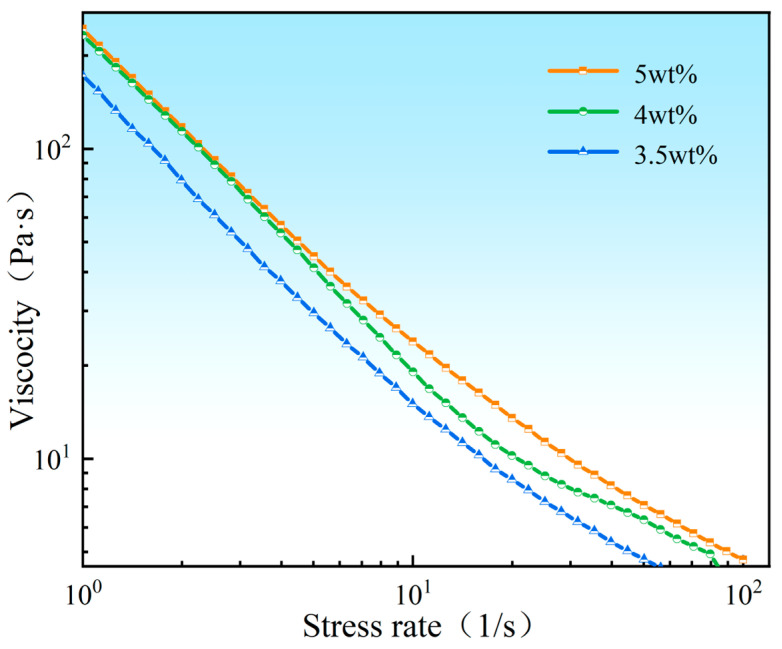
Apparent viscosity changes in three kinds of cellulose composite inks as a function of shear rates: 5 wt%, orange line; 4 wt%, green line; and 3.5 wt%, blue line.

For DIW 3D printing, the rheological properties of the ink and the printing parameters are two key factors [39,40]. Figure 3 shows the viscosity changes in three different composite inks upon the change in shear rate, and the three inks all exhibit clear shear-thinning behavior. At a shear rate of s^−1^, the viscosity of the 5 wt% formulation is 243.58 Pa·s, the viscosity of the 4 wt% formulation is 232.46 Pa·s, and the viscosity of the 3.5 wt% formulation is 174.01 Pa·s. When the shear rate increases to 10 s^−1^, the viscosity of the 5 wt% formulation is 23.91 Pa·s, the viscosity of the 4 wt% formulation is 19.04 Pa·s, and the viscosity of the 3.5 wt% formulation is 13.64 Pa·s. When the shear rate reaches 100 s^−1^, the viscosity of the 5 wt% formulation is 4.71 Pa·s, and the viscosity of the 4 wt% formulation is 4.04 Pa·s. As the shear rate increases, the viscosity of the different composite inks gradually decreases, all exhibiting typical shear-thinning behavior. The viscosity decreases with the increase in shear rate, which is favorable for the DIW process. Low viscosity at a high shear rate was beneficial to the smooth extrusion of the composite inks, and high viscosity at a low shear rate helped maintain the shape of the printed structure [41]. The 5 wt% formulation shows a higher viscosity compared to that of the other two formulations at different shear strains. This process enables the execution of smooth 3D printing, while concurrently preserving the structural integrity of the printed object.

#### 3.2.2. Thixotropy

**Figure 4:**
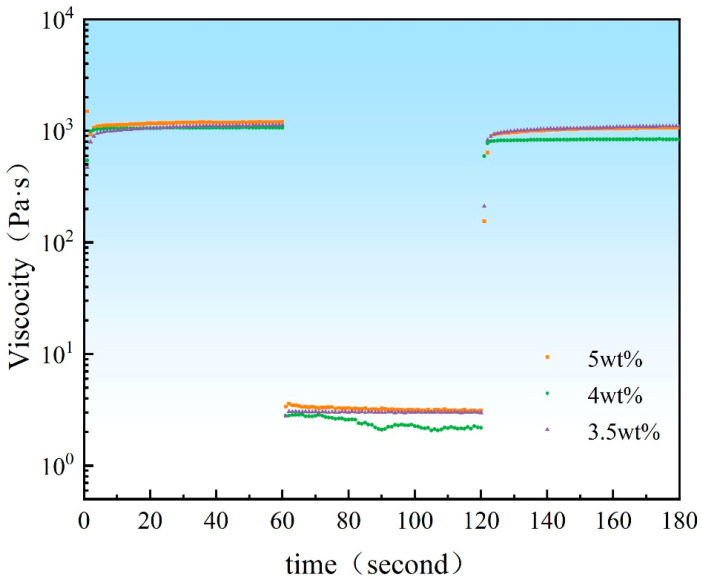
Dynamic viscosity changes of different composite inks before and after the printing process: 5 wt%, orange squares; 4 wt%, green circles; 3.5 wt%, purple triangles.

Figure 4 illustrates the viscosity changes in different composite inks before and after the printing process, covering three stages, i.e., before printing, during printing, and after printing. The results suggest that during the transition from low speed to high speed shear rate and back to low speed in the printing process, with no obvious time lag, the viscosity of the composite inks generates a trend of viscosity decreasing from high to low, followed by an increase after printing. Furthermore, the viscosity values after the transition from high speed back to low speed change rapidly, with values similar to those obtained at the initial low speed. This confirms that the composite inks show good shape retention after printing, which is beneficial for obtaining a well-defined 3D structure after printing.

#### 3.2.3. Herschel–Bulkley Fluid Model

Based on the rheological performance and viscosity analysis, we selected 5 wt% composite cellulose for the Herschel–Bulkley fluid model calculations. We evaluated the relationship between shear stress and shear rate for the 5 wt% composite ink using the Herschel–Bulkley fluid model for validation.

**Figure 5:**
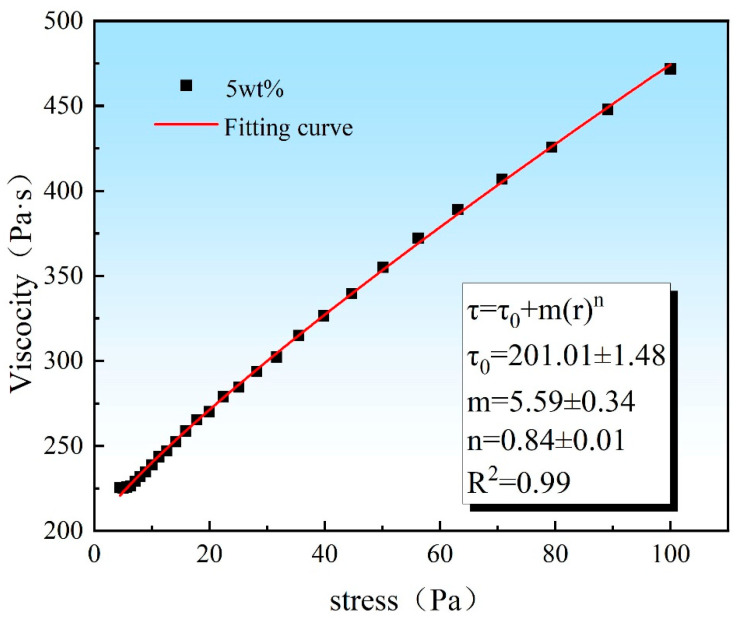
The relationship between shear stress and shear rate of the 5 wt% composite ink, fitting the Herschel–Bulkley Fluid model.

The shear stress–shear rate curve was fitted using the Herschel–Bulkley fluid model (Equation (1)), as shown in Figure 5. The relationship between the shear stress and shear rate for the 5 wt% composite ink aligns well with the Herschel–Bulkley fluid model. The results are *τ*_0_ = 201 Pa, m = 5.6, and n = 0.8. The static shear stress (*τ*_0_ = 201 Pa) means that the ink undergoes shear thinning behavior only when suffering from a shear stress higher than 201 Pa. The power law index (n = 0.8) is smaller than 1, meaning that the ink exhibits the characteristic of shear thinning. The consistency index (m = 5.6) is greater than 1, meaning that the ink can retain it shape after deposition. The results of the evaluation of the printability of the ink, based on the fitted formula, show that the ink exhibits obvious Bingham pseudoplasticity [42] and is easy to extrude from the nozzle; it can also maintain its shape and support subsequent layers.

#### 3.2.4. Oscillatory Strain Amplitude

**Figure 6:**
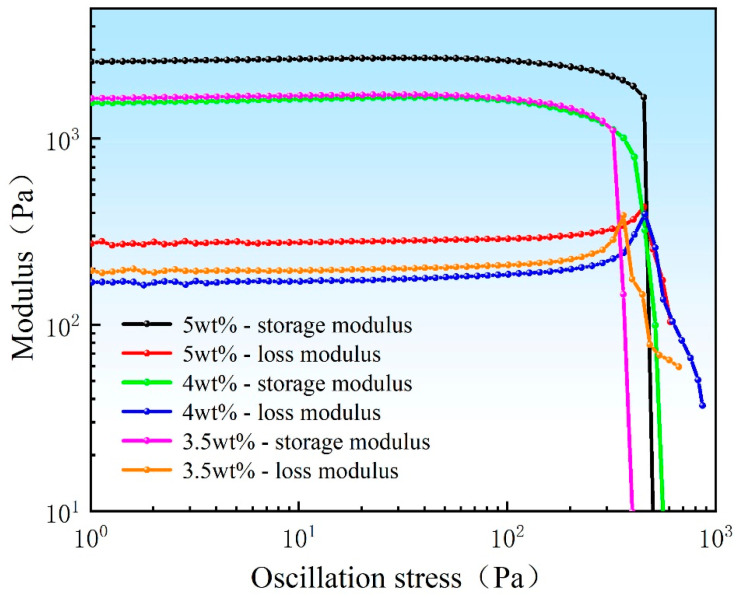
The composite inks’ loss modulus and storage modulus as a function of oscillation strain amplitude.

The oscillatory strain amplitude plays a critical role in identifying the transition of the ink from a solid to a liquid state. At low oscillatory strain, the storage modulus (G′) is greater than the loss modulus (G″), indicating that the ink exhibits solid-like behavior with strong elastic characteristics. At high oscillatory strain, G′ is less than the loss modulus G″, indicating that the ink exhibits liquid-like behavior, dominated by viscous characteristics [43]. Figure 6 presents the G″ and G′ of different composite ink formulations. In the figure, the black and red dashed lines represent G′ and G″ for the 5 wt% formulation, the green and blue dashed lines represent G′ and G″ for the 4 wt% formulation, and the purple and orange dashed lines represent G′ and G″ for the 3.5 wt% formulation. It can be observed that the yield stress for the 5 wt%, 4 wt%, and 3.5 wt% formulations are 470 Pa, 452 Pa, and 342 Pa, respectively. With the increase in composite cellulose content, the yield stress gradually increases, indicating that at higher concentrations of composite cellulose, the ink requires greater stress to transition from solid-like behavior to liquid-like behavior. This behavior is crucial for 3D printing applications, where the ink needs to maintain its shape during the printing process and flow smoothly when extruded [44].

#### 3.2.5. Solid–Liquid Distribution

The intersection point of the loss modulus and the storage modulus curves is crucial for identifying the transition of the composite ink from solid-like to liquid-like behavior. The appearance of this intersection marks the transition of the ink from elastic behavior to viscous behavior, which in turn affects its flow properties and printability [45]. To further assess the extrusion performance of the composite ink, it is necessary to calculate the nozzle stress distribution [38,46], as shown in Equation (2), in order to more accurately evaluate the solid–liquid phase transition of the ink during actual printing.

(2) $$\tau =\Delta P\times \frac{r}{2L}$$

where *P* is air pressure provided by the air compressor, *r* is the nozzle diameter, and *L* is the nozzle length.

**Figure 7:**
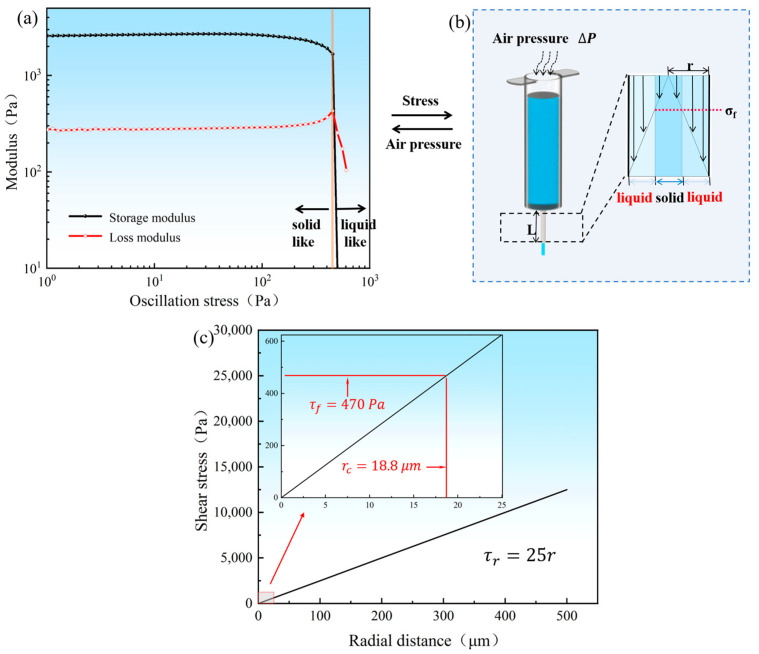
(**a**) The solid–liquid distribution of 5 wt% composite ink; (**b**) the nozzle stress distribution; (**c**) the critical radius for the transition from solid-like to liquid-like behavior.

Based on our printing parameters (*P* = 600 Kpa, *L* = 1.2 cm), the solid–liquid distribution for the 5 wt% composite ink is shown in Figure 7. For the 5 wt% composite ink, the intersection point of the loss modulus and storage modulus curves occurs at an oscillation stress of 470 Pa (Figure 7a). Before 470 Pa, the composite ink extruded from the nozzle during the printing process behaves like a solid, while after 470 Pa, the ink behaves like a liquid during extrusion. The nozzle stress distribution calculation formula indicates that the critical radius for the transition from solid-like to liquid-like behavior occurs at 18.8 μm (Figure 7c). When the nozzle diameter is larger than 18.8 μm, the composite ink behaves like a liquid, and the extruded ink lines from the nozzle are more uniform [47].

### 3.3. Porosity

To process a 1 m^3^ cubic mesh cellulose composite aerogel using DIW and in situ freezing techniques, with a density of 27.5 mg/cm^3^, the porosity of the composite aerogel can be calculated using its mass and volume, as shown in the following equation [48]:

(3) $$P=\frac{\left(\rho V-M\right)}{\rho V}\times 100$$

where *P* (%) is defined as porosity, *ρ* (g/cm^3^) is the density of the material (27.5 mg/cm^3^), *V* (cm^3^) is the volume of the scaffold (1 cm^3^), and *M* (g) is the measured weight of the scaffold.

This method combines the unique structural characteristics of cellulose composites with their processing techniques, enabling the effective characterization of the microporous structure and properties of the aerogel.

The calculated porosity of the composite aerogel is 98%.

### 3.4. Pore Morphology

**Figure 8:**
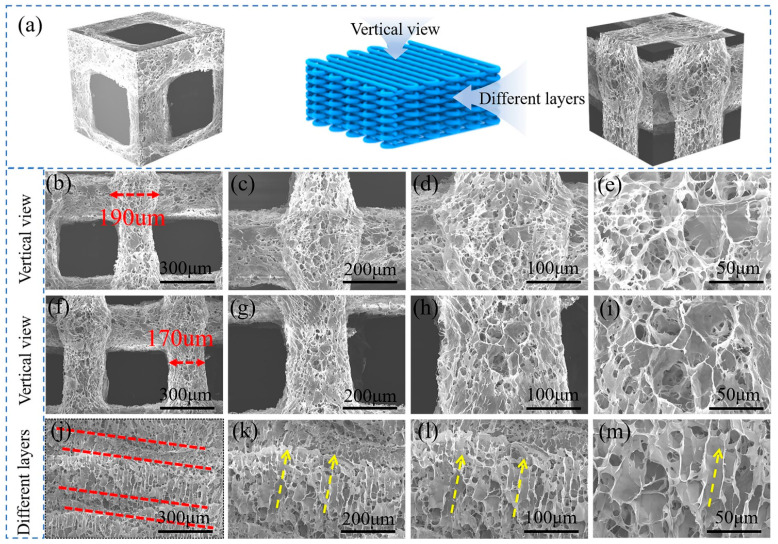
(**a**) Overall schematic view of the aerogel design and layer-by-layer structure; (**b**–**i**) SEM images of top view aerogels at different magnifications; (**j**–**m**) SEM images of side view of multiple layers of aerogels at different magnifications, showing aligned pores along the freezing direction.

By adjusting the material extrusion ratio and controlling the substrate temperature, the controllable construction of the 3D-printed cellulose composite aerogel deposition state was achieved. Figure 8 shows the SEM images of the 5 wt% cellulose derivative composite aerogel. Figure 8b–i shows the top view of the cellulose composite aerogel, while Figure 8j–m presents the layered structure of the cellulose composite aerogel at different levels. The results show that during the printing process, the extruded composite ink lines are relatively uniform, with line diameters ranging from 170 μm to 190 μm (Figure 8b,f). After the freeze-drying and refreezing process, the cellulose composite aerogel exhibits densely packed pores. SEM images from different layers reveal that the pores are oriented vertically upward. This is primarily due to the low substrate temperature control. During the sublimation process of ice crystal formation after refreezing, the gas sublimates upwards through the cellulose composite aerogel, resulting in the vertical pore channels observed in the structure [49,50].

### 3.5. Compression Properties

**Figure 9:**
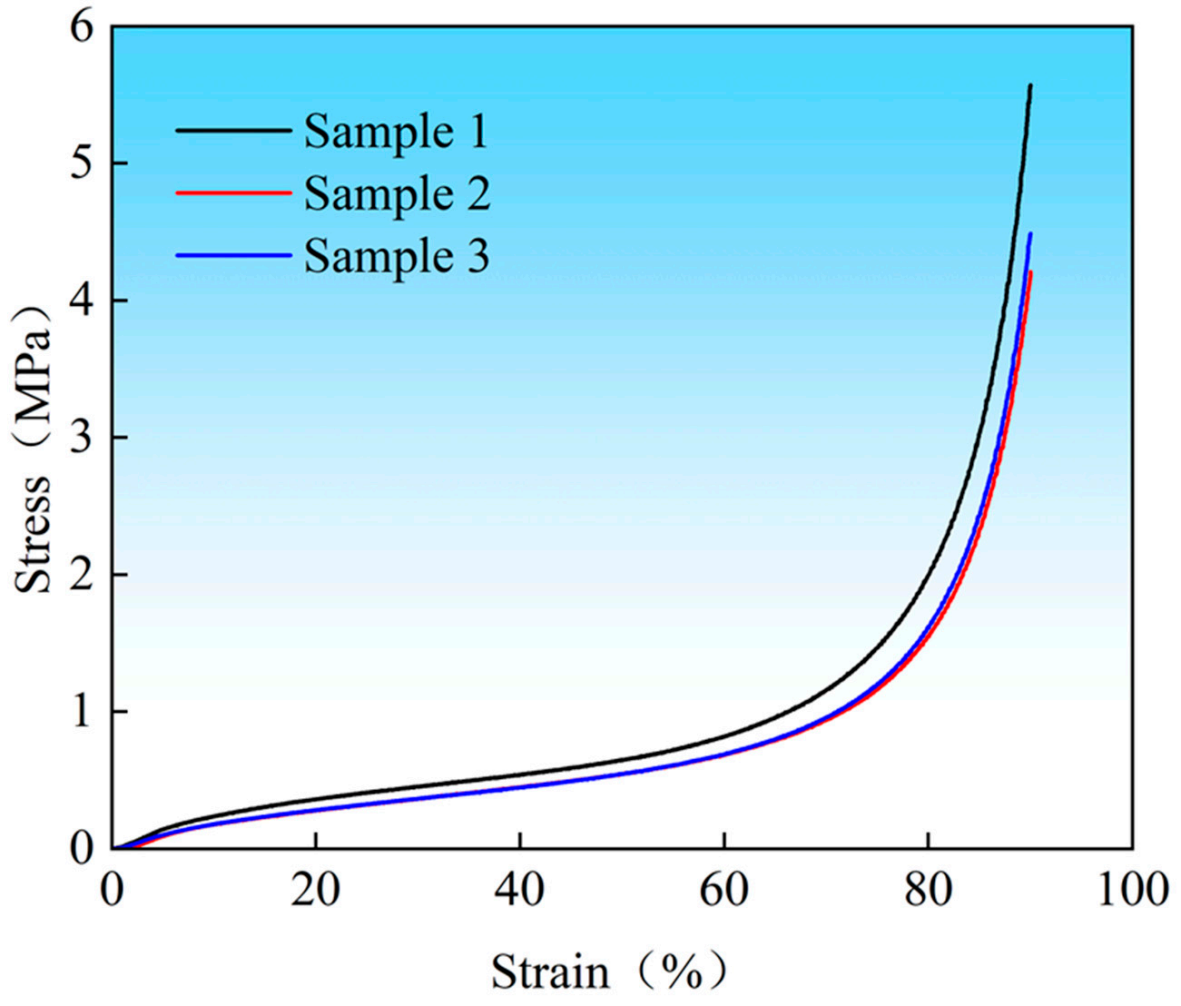
The compressive stress–strain curves.

The compressive stress–strain curves of all three sets of specimens were 3D printed with 5 wt% composite ink, since the 5 wt% formulation exhibits the best printability, as shown in Figure 9. The three curves are very close to each other over most of the strain range, indicating that our printing approach is very stable in producing each specimen, and that the compression test was conducted in a stable manner. The slight difference among the curves of the three samples in the high strain region may be related to the printing fluctuations during the printing process. The stress–strain of the specimen is divided into three stages. (1) When the strain is 0–10%, the initial stage of the specimen before the yield point is the linear elastic deformation stage. (2) When the strain is 10–60%, a relatively flat region is presented due to the plastic yielding of the specimen. (3) At strains greater than 60%, the curve becomes steeper as the specimen microstructure collapses and contracts. When the compressive strain of the specimen is loaded to 60%, the compressive stress of the specimen is 0.82 MPa. Comparing the compressive stresses of the CNF porous structure specimens and the 3D printed CNF specimens [51,52], the compressive stresses of the 5 wt% composite ink specimens were increased. It is demonstrated that the 3D printing of 5 wt% composite inks helps to improve their mechanical properties.

## 4. Discussion

In this study, we explore the 3D printing technology of cellulose-based aerogels with the aim of addressing the challenges they face in the conventional 3D printing process and proposing innovative solutions to improve printing and shape fixation, resolution, and structural performance.

A single cellulose suspension exhibits gel-like properties that make it unsuitable for DIW 3D printing. Its low viscosity results in an inability to maintain a complete shape during printing. The printed structure is prone to deformation or collapse, which limits its geometric complexity and resolution, making it difficult to achieve high-precision 3D printing [23,24].

To optimize the formulation, we have added three key ingredients, TEMPO-CNFs, TEMPO-CNC, and CMC. The electrostatic repulsion between them is used to form a stable and homogeneous suspension, which increases the viscosity and structural stability of the ink, improves printability and dimensional stability, and makes the formulation more suitable for DIW printing. The inks with different formulations show significant differences in their rheological properties [47,53,54].

The introduction of an in situ freezing stage into the printing process allows the deposited ink to freeze within seconds, enabling rapid molding. This reduces ink shrinkage and distortion and ensures the dimensional shape fixation of the printed portion. It also improves the fidelity of the printed structure and effectively maintains the structural integrity of the printed component. The freeze-drying process creates a multi-scale pore structure in the printed samples. This increases the specific surface area of the material and improves the adsorption properties of the aerogel. This method provides aerogels with favorable attributes for environmental and biomedical applications [55]. Moreover, printing accuracy and shape fixation are very important for 3D printing, and we will clarify the research regarding accuracy and shape fixation in 3D printing in our future work.

In view of the biomedical applications of cellulose-based materials, we need to further investigate the biocompatibility and biodegradability of the printed aerogel. Then, these DIW 3D-printed high-resolution cellulose-based aerogels will meet the stringent requirements of biomedical applications for aerogels and expand their use in tissue engineering, drug release, and other fields [56].

## 5. Conclusions

In this work, we have developed a cellulose composite ink based on TEMPO-CNFs, TEMPO-CNC, and CMC and demonstrated a direct ink writing technique for preparing a stable three-dimensional architecture. A few key conclusions are summarized below:

1. The cellulose composite inks with different ratios were prepared through a mixing and centrifugation method. The shear thinning behavior and Bingham-pseudoplasticity properties make the 5 wt% ink suitable for high-precision extrusion printing for three-dimension printing.
2. With rational design, the composite inks TEMPO-CNFs, TEMPO-CNC, and CMC neutralizing hydrogen bonds with electrostatic repulsion to achieve optimal dispersion.
3. TEMPO-CNC increased the mechanical stability of the cellulose composite ink during printing.
4. CMC regulates the viscosity of the composite cellulose and maintains the shape of the material during the printing process.
5. Using an in situ freezing strategy, the material is frozen in situ during the 3D printing process, resulting in 3D structures with a resolution of less than 200 μm (170 μm), with finer structures. Printing and shape fixation is improved because the printed shape is the same as that of our original design model, and the composite ink is able to accurately print a 1 cm^3^ shape. The printed 3D samples can exhibit a compressive stress of up to 0.82 MPa, with a porosity of more than 98%.
6. These architectures printed using cellulose composite ink not only display regular, square, through-hole structures via a 3D-printed design, but also exhibit more irregular micropores generated after freeze-drying. The multi-level pore design, providing more surface areas, and the hydrophilic nature of the material offer potential for use in biomedical areas such as the printing of customized point-of-care hemostatic materials.

## Figures and Tables

**Table 1:** Composite ink main information.

Material Code	Concentration	Polymerization Degree	Carboxyl Content	Average Length	Supplier
TEMPO-CNF	3 wt%	110	1.86 mmol/g	0.8–1.2 μm	Wood Spirit Biotechnology Co., Ltd. (Tianjin, China)
TEMPO-CNC	10 wt%	100	2.03 mmol/g	120 nm	Wood Spirit Biotechnology Co., Ltd. (Tianjin, China)
CMC	99.56 wt%	/	3.20 mmol/g	/	Aladdin Biochemical Technology Co., Ltd. (Shanghai, China)

**Table 2:** The formulation of cellulose composite ink used for printing.

Formulation Type	Solid Contents	TEMPO-CNF	TEMPO-CNC	CMC	DI Water
1	5 wt%	2.5 wt%	1.5 wt%	1 wt%	0
2	4 wt%	2.5 wt%	1.5 wt%	0	1 mL
3	3.5 wt%	2.5 wt%	0	1 wt%	9 mL

**Table 3:** Instruments and conditions required at different stages.

Type	Experimental Phase	Instruments	Temperature	Time
a	gelation	stirrer and centrifuge	25 °C	0.5 h
b	3D printing	dispenser	−10 °C	0.5 h
c	in situ freezing	semiconductor Peltier stage	−30 °C	24 h
d	freeze drying	LGJ-10 Freeze Dryer	−60 °C	48 h

**Table 4:** Equipment and conditions required for different experiments.

Type	Measurement Properties	Equipment	Conditions	Sample Code
1	Zeta potential	Zetasizer Nano ZS90	Dilution with DI water	5 wt%/4 wt%/3.5 wt%
2	Rheological properties	MCR 302	Different shear rates	5 wt%/4 wt%/3.5 wt%
3	Pore size	FE-STEM SU9000	10.0 KV	5 wt%
4	Compression test	E43.104	0.5 mm/min	Sample 1, 2, 3

## Data Availability

The authors confirm that the data supporting the findings of this study are available within the article.
